# *PIK3CA* exon9 mutations associate with reduced survival, and are highly concordant between matching primary tumors and metastases in endometrial cancer

**DOI:** 10.1038/s41598-017-10717-z

**Published:** 2017-08-31

**Authors:** Siv Mjos, Henrica M. J. Werner, Even Birkeland, Frederik Holst, Anna Berg, Mari K. Halle, Ingvild L. Tangen, Kanthida Kusonmano, Karen K. Mauland, Anne M. Oyan, Karl-Henning Kalland, Aurélia E. Lewis, Gordon B. Mills, Camilla Krakstad, Jone Trovik, Helga B. Salvesen, Erling A. Hoivik

**Affiliations:** 1 Centre for Cancer Biomarkers, Department of Clinical Science, University of Bergen, Bergen, Norway; 2 Department of Gynecology and Obstetrics, Haukeland University Hospital, Bergen, Norway; 3 Centre for Cancer Biomarkers, Department of Clinical Medicine, University of Bergen, Bergen, Norway; 4 Department of Pathology, Haukeland University Hospital, Bergen, Norway; 5 Computational Biology Unit, University of Bergen, Bergen, Norway; 6 Bioinformatics and Systems Biology Program, School of Bioresources and Technology, King Mongkut’s University of Technology Thonburi, Bangkhuntien, Bangkok, Thailand; 7 Department of Microbiology, Haukeland University Hospital, Bergen, Norway; 8 Department of Molecular Biology, University of Bergen, Bergen, Norway; 9 Department of Systems Biology, University of Texas MD Anderson Cancer Center, Houston, Texas USA

## Abstract

Mutations of the phosphoinositide-3-kinase (PI3K) catalytic subunit alpha gene (*PIK3CA*) are frequent in endometrial cancer. We sequenced exon9 and exon20 of *PIK3CA* in 280 primary endometrial cancers to assess the relationship with clinicopathologic variables, patient survival and associations with *PIK3CA* mRNA and phospho-AKT1 by gene expression and protein data, respectively. While *PIK3CA* mutations generally had no impact on survival, and were not associated with clinicopathological variables, patients with exon9 charge-changing mutations, providing a positive charge at the substituted amino acid residue, were associated with poor survival (p = 0.018). Furthermore, we characterized *PIK3CA* mutations in the metastatic setting, including 32 patients with matched primary tumors and metastases, and found a high level of concordance (85.7%; 6 out of 7 patients), suggesting limited heterogeneity. *PIK3CA* mRNA levels were increased in metastases compared to the primary tumors (p = 0.031), independent of *PIK3CA* mutation status, which rather associated with reduced *PIK3CA* mRNA expression. *PIK3CA* mutated tumors expressed higher p-AKT/AKT protein levels, both within primary (p < 0.001) and metastatic lesion (p = 0.010). Our results support the notion that the PI3K signaling pathway might be activated, both dependent- and independently of *PIK3CA* mutations, an aspect that should be considered when designing PIK3 pathway targeting strategies in endometrial cancer.

## Introduction

Endometrial cancer is the most frequent female pelvic malignancy in industrialized countries and the incidence is increasing^1, 2^. The most common basis for determining prognosis and risk for developing systemic disease is the distinction into type I and II, based on histologic type and grade^3^. Molecular studies attempt to improve this classification since up to 20% of the type I cancers will recur, while 50% of the type II cancers do not recur^2^. Such characterization has shown that type I cancers are more often mutated in *Homo sapiens* v-Ki-ras2 Kirsten rat sarcoma viral oncogene (*KRAS*), fibroblast growth factor receptor 2 (*FGFR2*) and phosphatase and tensin homology (*PTEN*), and tend to be microsatellite instable and estrogen- and progesterone receptor positive^2, 4^. Type II cancers, that often display hormone receptor loss, have altered expression of p53 and p16 and *HER2* overexpression and amplification^2, 4^. In addition, recent comprehensive genetic profiling has refined the molecular classification of endometrial carcinomas, reflecting clinical phenotypes^2, 5^. While these differences have prognostic value, they have so far not been utilized therapeutically^4, 6^.

The phosphoinositide 3-kinase (PI3K) signaling pathway is critical in maintaining a normal balance between cell survival and apoptosis. Activating alterations in the PI3K pathway are frequently found in cancer, and deregulation of components of this pathway is associated with carcinogenesis^7–10^. Class IA PI3Ks are heterodimers consisting of one catalytic (p110α p110β and p110δ and one regulatory subunit (p85α p85β and p55γ. p110α is encoded by the PI3K catalytic subunit alpha gene *PIK3CA* and is the most frequently altered isoform in human cancer^8, 11^. Under normal conditions, the PI3K pathway is activated through transmembrane tyrosine kinase growth factor receptors (RTK) or by G-protein coupled receptors, relieving the inhibitory activity of the regulatory subunit, allowing the catalytic subunit to phosphorylate phosphatidylinositol(4,5)*-*bisphosphate (PIP_2_) to generate phos-phatidylinositol(3,4,5)-triphosphate (PIP_3_). The reverse process is regulated by *PTEN* that is able to dephosphorylate PIP_3_ to PIP_2_. PIP_3_ acts as a docking molecule for the serine/threonine kinases phosphoinositide-dependent kinase 1 (PDK1) and AKT/protein kinase-B (PKB), enabling phosphorylation and activation of AKT1 (p-AKT) at the T308 residue^11, 12^. Phosphorylated AKT (p-AKT) leads to activation of mammalian target of rapamycin (mTOR) complex and increased protein synthesis of its effector p70S6K and phosphorylation of ribosomal S6 protein^11^.

In primary endometrial cancer lesions, *PIK3CA* is the second most frequently significant mutated gene after *PTEN*, with a frequency of 53% according to comprehensive genomic profiling including whole exome sequencing performed by The Cancer Genome Atlas (TCGA)^5, 13, 14^. Mutations at exon9 hotspot residues, p.E542, p.E545 and p.Q546, may cause a charge-plus change; that is a change from negatively charged glutamic acid (E) (or glutamine [Q], uncharged) to positively charged Lysine (K), and lead to an abrogation of the interaction with the regulatory subunit p85α (encoding gene: *PIK3R1*) and hence, relieving the inhibitory effect on p110α^15–20^. In contrast to the characterization performed on primary tumors, PI3K/AKT alterations in metastases or recurrences are not well described.

Despite the fact that most *PIK3CA* mutations lead to an overactive enzyme with growth promoting properties^16, 19^, the literature is inconsistent when describing the prognostic impact of *PIK3CA* mutations and in particular regarding the different classes of *PIK3CA* mutations on patient survival in different cancer types^21–23^. There is conflicting evidence of the effect of *PIK3CA* mutations on clinical variables in endometrial cancer, although relations to depths of myometrial infiltration and differentiation grade have been reported^24, 25^.

The purpose of this study was to analyze the impact of *PIK3CA* mutations, on survival and clinical characteristics, and their relation with additional markers in the PI3K pathway, including assessment of AKT1 activation status (p-AKT_T308/AKT ratio), focusing on patients with systemic disease. Next, we wanted to assess the degree of concordance of *PIK3CA* hotspot mutations in a set of 32 matched pairs of primary endometrial cancers and metastases.

## Results

### The occurrence of *PIK3CA* helical and kinase mutations in primary endometrial cancer

**Figure 1:**
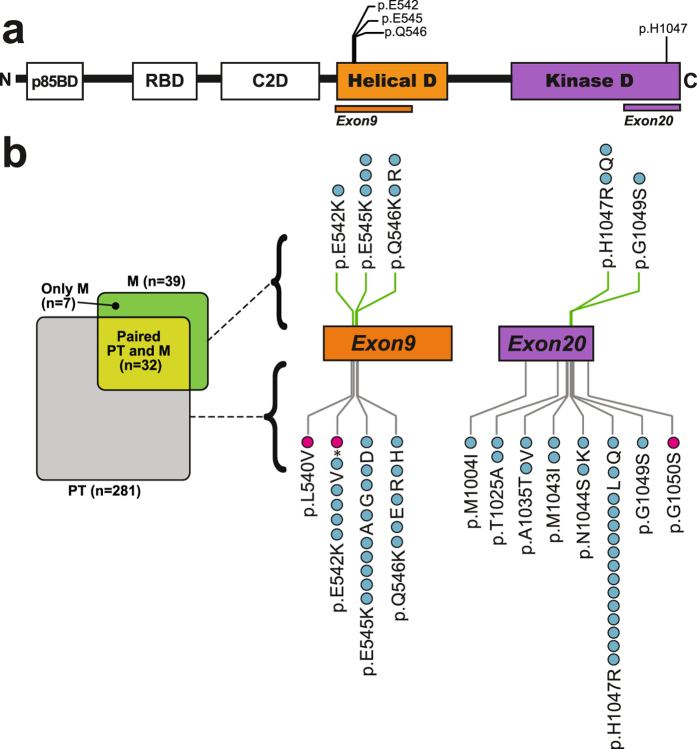
Occurrence of mutations within exon9 and exon20 of the *PIK3CA* gene in endometrial cancer. (**a**) Overview of *PIK3CA* gene, encoding the p110α protein, a catalytic subunit of PI3K class IA proteins. Protein regions corresponding to exon9; helical domain and exon20; kinase domain, are shown with known mutational hotspot positions indicated on top. Abbreviations: p85BD; p85α regulatory subunit binding domain, RBD; RAS-binding domain, C2D; protein-kinase-C-homology-2 domain, Helical D; helical domain, Kinase D; kinase domain. (**b**) Overview of primary tumor (PT) and metastasis (M) sample sets and overlapping matching PT and M paired sample set (left side). Number of patients is denoted in parenthesis. Twenty-four unique mutations were observed in primary and (top-side of the exons) or in a primary tumor (under-side of the metastatic lesions by Sanger sequencing. Mutations found in primary and metastatic lesions are indicated at each side of the exon. Each dot represents a mutation in either a metastatic lesion exons). The blue dots represent a mutation that have been previously reported in the Catalogue of Somatic Mutations in cancer (COSMIC, v79) or The Cancer Genome Atlas (TCGA) databases, while the pink dots represent novel mutations not previously found in any cancer types or not seen in endometrial cancer. Three primary tumors revealed double mutations: p.E545A/E545D, p.E542V/E542*(STOP), p.Q546R/G1049S.

To investigate the functional consequences of mutations located in the helical membrane association domain and/or in the kinase domain of *PIK3CA*, we sequenced exon9 and exon20, respectively. The mutation rate was 15.7% (Fig. 1 and Supplementary Table S1), in agreement with earlier and similar reports of *PIK3CA* mutations (range 12–39%; by Sanger sequencing)^24, 25, 35–37^. 25 different missense mutations and one nonsense mutation predicted to affect 24 different amino acids (AAs) in the *PIK3CA* encoded protein (p110α) were identified (Fig. 1b and Supplementary Table S2). Specifically, 21 of the mutations were found within four AAs located in the helical domain, while 26 mutations were focused at eight AAs in the kinase domain in the primary tumors. Of these, two cases had two mutations in exon9, and one case had mutations in both exons 9 and 20. In total, we identified 47 mutations, distributed over 44 cases with primary tumors.

Four positions on *PIK3CA* were more frequently mutated and considered hotspot sites in agreement with other studies; p.E542, p.E545, p.Q546 and p.H1047, with 6, 9, 5 and 15 mutations, respectively (Fig. 1 and Supplementary Table S2)^24, 25, 38^. Mutations within these AAs accounted for 75% of all the mutations we detected in primary tumors, comparable to the rate in TCGA and Catalogue of Somatic Mutations in cancer (COSMIC)^13, 14, 39^ (74.4% and 75%, respectively; Supplementary Table S1).

In addition to hotspot mutations, we detected p.E542*(STOP), a *PIK3CA* mutation never observed in any cancer and two mutations that had not previously been reported in endometrial cancer; p.L540V and p.G1050S (Fig. 1b and Supplementary Table S2)^5, 13, 14, 39^.

### Relation of *PIK3CA* mutations to clinicopathological variables, survival and markers of the PI3K pathway

**Figure 2:**
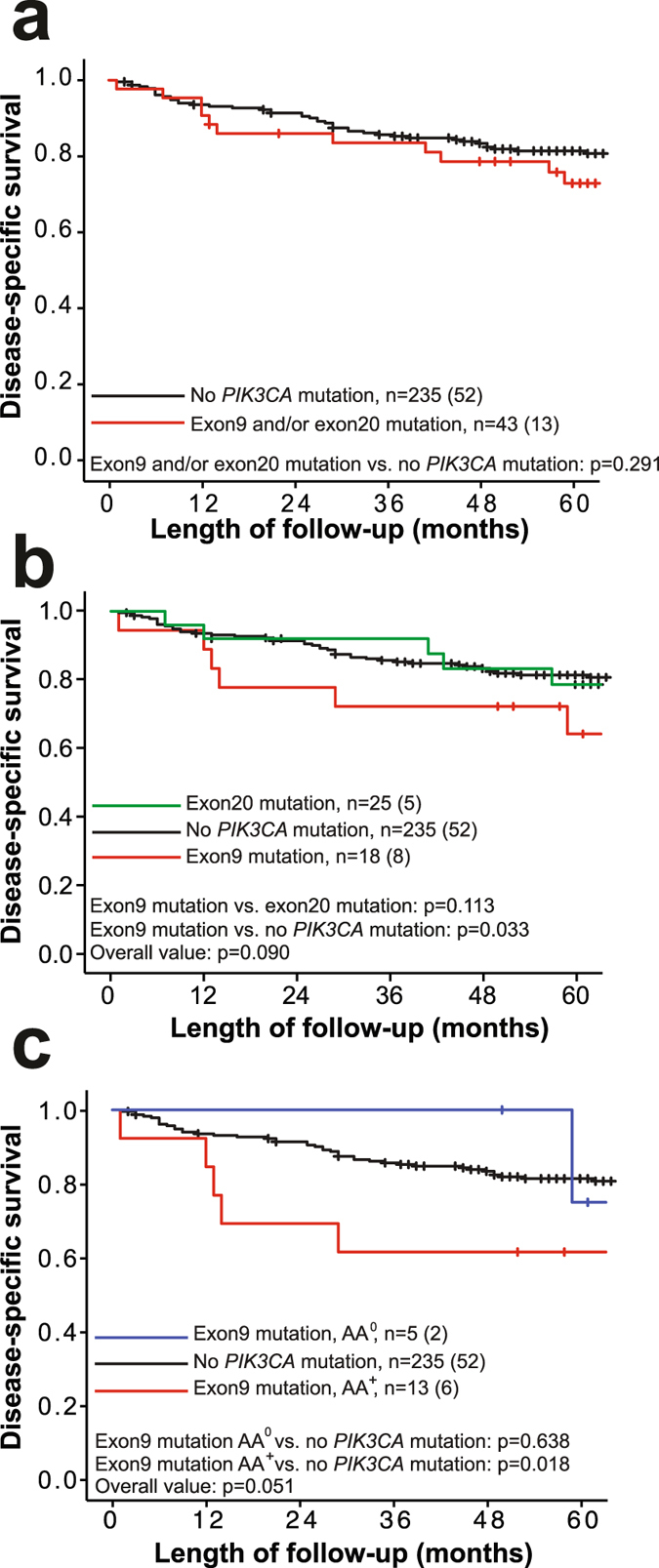
Disease-specific survival according to the *PIK3CA* mutation status in endometrial cancer. (**a**) *PIK3CA* mutation (exon9 and/or exon20) compared to no mutation detected. (**b**) *PIK3CA* exon9 mutations compared to *PIK3CA* exon20 mutations or non-mutated. (**c**) *PIK3CA* exon9 mutation leading to a change from negative to positive charge at affected amino acids (AA^+^), compared to mutation not leading to change of charge (AA°) and compared to no mutation. Curves are plotted by the Kaplan-Meier method; n = number of cases in each category followed by number of disease specific deaths, with comparison of survival between categories using log-rank (Mantel-Cox) test.

The *PIK3CA* mutations in primary tumors were not associated with any of the clinicopathologic variables or biomarkers investigated (Table 1 and Supplementary Table S3). *PIK3CA* mutations did not influence disease specific endometrial carcinoma survival (Fig. 2a). However, structural analyses of *PIK3CA* mutations have elucidated that helical and kinase mutations induce gain-of-function of p110α through different mechanisms^7, 40^. When stratifying according to localization of the *PIK3CA* mutations (Fig. 2b), patients with exon9 mutations in the helical domain, tended to have a worse outcome compared to patients with exon20 mutations of the kinase domain (p = 0.113) or patients with no *PIK3CA* mutations detected (p = 0.033).

**Table 1:** Comparison of clinicopathologic variables and *PIK3CA* mutation status in 280 primary endometrial carcinomas assessed by Sanger sequencing.

*PIK3CA* mutation	*Total cases n*	*No*	*Yes*	p-value^a^
Variable		n (%)	n (%)	
Age	280			0.4
<66		123 (86.6)	19 (13.4)	
≥66		114 (82.6)	24 (17.4)	
BMI	271			0.6
<30		153 (83.6)	30 (16.4)	
≥30		76 (86.4)	12 (13.6)	
FIGO stage	280			0.8
I-II		191 (84.9)	34 (15.1)	
III-IV		46 (83.6)	9 (16.4)	
Myometrial infiltration	278			0.1
<50% infiltration		140 (88.1)	19 (11.9)	
≥50% infiltration		96 (80.7)	23 (19.3)	
Lymph node metastasis	235			0.9
No		170 (84.2)	32 (15.8)	
Yes		28 (84.8)	5 (15.8)	
Histologic type	280			0.4
Endometrioid		195 (85.5)	33 (14.5)	
Non-Endometrioid		42 (80.8)	10 (19.2)	
Grade	277			0.6
High-medium		149 (85.6)	25 (14.4)	
Low		85 (83.3)	17 (16.7)	
DNA ploidy	231			0.4
Diploid		147 (86.0)	24 (14.0)	
Aneuploid		49 (81.7)	11 (18.3)	
ERα	273			0.8
Positive		175 (85.3)	31 (15.0)	
Negative		56 (83.6)	11 (16.4)	
PR	276			0.6
Positive		174 (84.1)	33 (15.9)	
Negative		60 (87.0)	9 (13.0)	

^a^The p-value was estimated using the Chi-square test. n = number of cases analyzed. Abbreviations: BMI; body mass index, FIGO; International Federation of Gynecology and Obstetrics. ERα; Estrogen receptor alpha. PR; progesterone receptor.

Mutations of the helical domain, encoded by exon9 were further stratified into two categories; those predicting a charge-plus alteration, (from negative to positive charged AA, herein denoted as AA^+^) and those mutations predicting not to cause such a change (AA°) (Supplementary Table S2). We added p.Q546R and p.Q546H to the group of AA^+^ previously described (p.E542K, p.E545K, p.Q546K)^16^, since these mutations also provide a change to a positively charged side chain of the substituted AA. Patients with AA^+^ mutations had poorer survival (p = 0.018) compared to patients with wild type *PIK3CA* or substitutions without charge-plus change (Fig. 2c). In multivariate survival analyses, the AA^+^ substitutions demonstrated an independent prognostic impact (p = 0.012, HR of 3.00, CI 1.27–7.07) when adjusting for important clinicopathologic variables in a multivariate Cox model (age, histologic type and grade; Supplementary Table S4). Further survival analysis based on these charge-positive substitutions showed reduced survival in non-endometrioid histologic type (p = 0.019; Supplementary Fig. S1a), but they did not in the endometrioid type tumors (Supplementary Fig. S1b). There was no correlation between exon9 mutations and histologic type when split into two groups (endometrioid and non-endometrioid; p = 0.749; Fisher’s Exact Test), nor with specific histologic subtypes listed in Supplementary Table S6 (p = 0.759; Pearson’s Chi-Square test). Similarly, exon9 AA^+^ cases were neither correlated to histologic types (endometrioid and non-endometrioid; p = 0.267, Fisher’s Exact Test, Supplementary Table S3) and nor to specific histologic subtypes (Supplementary Table S6, p = 0.499; Pearson’s Chi-Square test). Similar results were found when analyzing recurrence-free survival, where cases with initial metastatic disease were excluded. Also, in this setting, exon9 mutations and exon9 AA^+^ mutations also provided significantly lower survival compared to non-mutated (Supplementary Fig. S2; p = 0.007 and p = 0.036, respectively).

As *PIK3CA* mutations may affect different targets in the PI3K signaling pathway, we investigated these mutations in relation to several molecular markers known to be associated with PI3K pathway activation or to poor prognosis in endometrial cancer (Supplementary Table S5); including protein expression of Stathmin, p85α and PTEN (immunohistochemistry; IHC), KRAS mutations, *PIK3CA* amplification (assessed by Fluorescent *in situ* hybridization; FISH) or *PIK3CA* mRNA expression (Agilent microarray)^7, 11, 24, 27, 30^. The *PIK3CA* mutations did not correlate with any of the abovementioned factors. In contrast, a significant correlation (p = 0.007) was revealed between *PIK3CA* mutations and high p-AKT/AKT expression (ratio of RPPA-values), indicative of PI3K pathway activation (Supplementary Table S5).

### Specific *PIK3CA* mutations are consistent from primary tumor to metastasis in paired tumor samples

**Figure 3:**
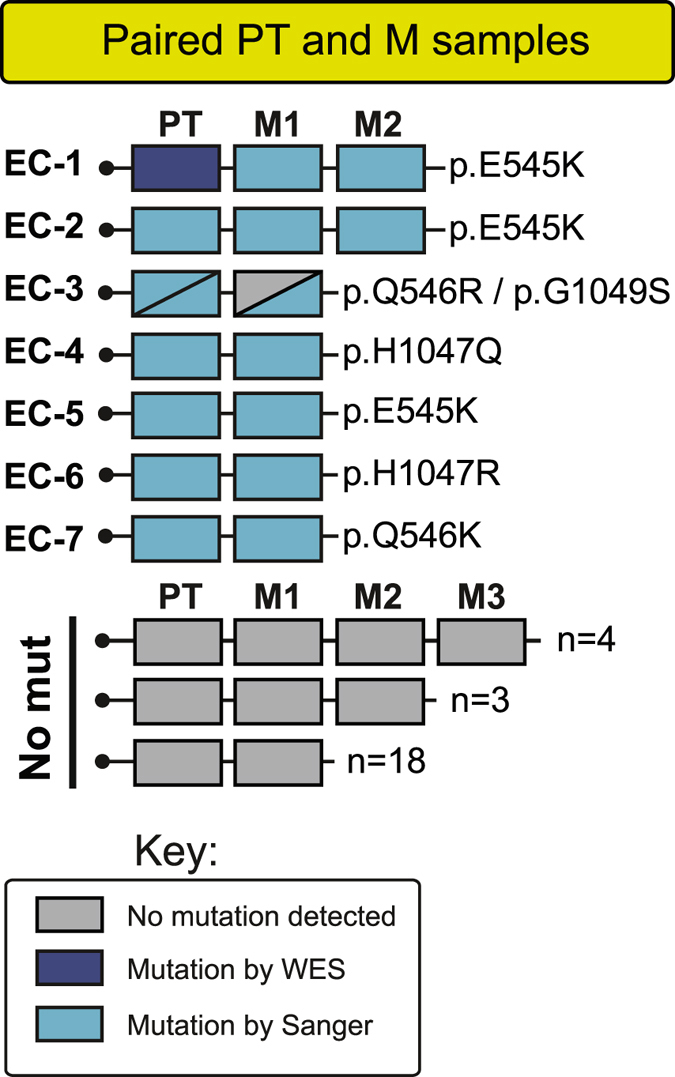
The specific *PIK3CA* mutations are concordant in the metastases compared with their paired corresponding primary endometrial cancer tumor. Most specific mutations in *PIK3CA* within primary tumors are detected in their corresponding metastasis counterpart within same patient. Key-box with color indications for mutation status and method used for detecting mutation. Abbreviations: PT; primary tumor, M1-3; unique individual metastases numbered for patients with multiple metastatic lesions, WES; whole exome sequencing.

*PIK3CA* mutation rates were similar for primary tumors that later metastasized (17.5%; n = 97) compared to those that did not metastasize (14.7%; n = 184). Within the unique set of primary tumors with corresponding metastases (n = 32), the mutation frequency in metastases (21.9%) was similar to the primary tumors (18.8%). Seven patients out of 32 with overlapping primary and metastatic lesions available for sequencing were found to harbor *PIK3CA* mutations (Fig. 3). The exact same mutation in the primary and corresponding metastatic lesion was detected in six out of the eight specific mutations detected in these cases. Two discordant cases presented a *PIK3CA* mutation only in one of the two types of lesions: Patient EC-1 presented the same mutation in two metastatic lesions that was not found in the primary tumor. Patient EC-3 had two *PIK3CA* mutations in the primary tumor, but only one of these mutations was detected in its corresponding metastasis (Fig. 3). Possible explanations of the mutation discordance between the primary tumor and the metastatic lesion for the two patients may be attributed to too low sensitivity of the applied sequencing method, tumor heterogeneity, or the content of tumor cells versus stromal contamination. High-throughput sequencing data has reported substantial intratumor heterogeneity as well as intertumor heterogeneity between primary tumor and metastasis^41, 42^. Interestingly, in an unrelated *in depth* whole exome sequencing study, we were able to detect the “missing” mutation also in the EC-1 primary tumor^43^. Thus, the final concordance of *PIK3CA* mutations is 87.5% in our sample set.

### *RNF183* expression is significantly upregulated in *PIK3CA* mutated primary tumors

We exploited our array-derived mRNA expression data for primary tumors with accompanying mutational status (n = 135), to search for differentially expressed genes between tumors that were *PIK3CA* mutated or not, through the Significance Analysis of Microarray (SAM) method. Applying the cutoffs as described in the methods section, we only found one gene; the E3 ubiquitin ligase ring finger protein 183 (*RNF183*), to be significantly differentially expressed between the two groups with a fold change of 3.7 (log2-scale) in the *PIK3CA* mutated tumors compared to those with no mutation detected. Thus, global mRNA expression was not noticeably different in *PIK3CA* mutated compared to non-mutated tumors.

### Metastatic lesions show increased *PIK3CA* mRNA expression independent of mutation status, and increased p-AKT/AKT protein levels dependent of *PIK3CA* mutations

**Figure 4:**
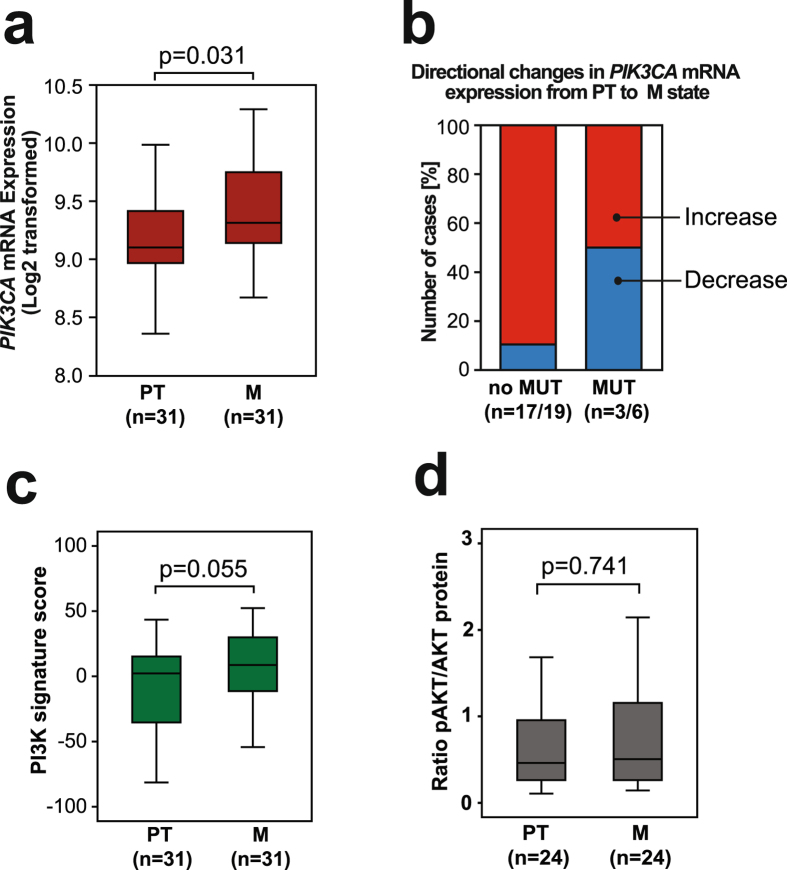
*PIK3CA* expression level increases from corresponding primary to metastatic lesions. (**a**) The *PIK3CA* mRNA expression level increases significantly from the primary tumors to metastatic lesions measured within same patient. (**b**) Binary directional shift in *PIK3CA* expression level in the metastasis compared to the primary tumor. The absence of mutation is most likely to increase *PIK3CA* expression towards metastatic state, while half of the cases with mutations also display reduced *PIK3CA* expression in the metastatic lesions. Number of cases is indicated as affected cases/total cases in each group. (**c**) The PI3K mRNA signature score defined by Gustafson *et al*.^29^ and (**d**) expression of p-AKT/AKT comparing the paired samples of corresponding primary and metastatic tumors. For simplicity, first metastatic lesion (M1) was used as comparison when multiple metastases were available. Statistical test: Mann-Whitney U test.

We investigated the mRNA expression of *PIK3CA*, and found it increased from primary tumor to paired metastases within paired samples (p = 0.031; Fig. 4a). This increase was mostly attributable to cases without mutations, as half of the patients with mutations in this paired set showed reduced expression levels considering directional *PIK3CA* mRNA changes from primary to metastatic lesion within same case (Fig. 4b). To elucidate the effect of *PIK3CA* mutations on *PIK3CA* expression, we investigated the subset of patients with both mutation and expression data available (n = 169; Supplementary Fig. S3a), and found that mutations significantly reduced the *PIK3CA* expression, but only in the metastases (p = 0.017). High *PIK3CA* expression in primary tumors alone was associated with significant poorer survival compared to cases with low expression (p < 0.001; Supplementary Fig. S1c).

We employed the PI3K signature score developed by Gustafson^29^, as a measure of PI3K pathway activation, on the set of paired primary and metastatic tumors, and found a tendency of increased PI3K activation in metastases (borderline significance of p = 0.055; Fig. 4c). The PI3K activation score seemed to be independent of the *PIK3CA* mutation status (Supplementary Fig. S3b). Measuring the p-AKT/AKT-ratio, with AKT acting downstream of *PIK3CA*, we found no significant differences between primary and metastatic lesions (Fig. 4d; RPPA data). However, a correlation between *PIK3CA* mutations and p-AKT/AKT was observed, with increased p-AKT/AKT in mutated compared to non-mutated *PIK3CA* tumors, both within primary tumors (p < 0.001) and within metastases (p = 0.010; Supplementary Fig. S3c).

We also investigated how the expression levels of these PI3K-pathway factors influenced cancer progression towards metastasis. The expression of *PIK3CA* mRNA increased concurrent with more invasive features within primary tumors (Supplementary Fig. S3d), comparing primary tumors that did not recur during follow-up to those that were diagnosed as metastatic at primary surgery (p = 0.005) or those that recurred later (p = 0.003). The PI3K signature score did not reach significance within these groups (Supplementary Fig. S3e). However, the p-AKT/AKT-ratio was higher in cases with no recurrence compared to those that were metastatic at primary surgery (p = 0.003; Supplementary Fig. S3f).

## Discussion

*PIK3CA* mutations are known to activate the PI3K pathway. Despite extensive genetic characterization, the impact of *PIK3CA* mutations on clinical outcome and their relation to clinical variables remains unsettled in many cancer types, including endometrial cancer^11, 16, 44^. Considering the high prevalence of *PIK3CA* mutations in endometrial cancer, and the unresolved impact in clinical and metastatic settings, we focused our study on mutations of *PIK3CA* ^5^.

In endometrial cancer, findings related to patient survival are contradictory and have suggested that *PIK3CA* mutations are favorable^45^, unfavorable^46^ or have neutral effect on patient survival^36, 47^.

In our data, the presence of a *PIK3CA* mutation, considering all *PIK3CA* mutations, had no impact on survival, possibly because mutations in different exons may act through different mechanisms^7, 40^. However, we found an association between *PIK3CA* exon9 mutations and poor outcome in this study, contrasting exon20 mutations that were associated with improved survival. Data supporting our findings, relating exon9 mutations and survival have been reported for non-small cell lung cancer, soft-tissue sarcomas and breast cancer^22, 48, 49^, wheremutations were associated with reduced survival.

Interestingly, we found that patients with exon9 charge-plus changing substitutions in the helical domain showed even poorer survival (p = 0.018; Fig. 2c). Activating mutations in the helical domain (hotspots p.E542, p.E545 and p.Q546) are positioned at an exposed surface and believed to have the same effect as PI3K activation through RTK; abrogating the inhibitory effect of regulatory subunit p85α on the p110α catalytic subunit by interfering with the nSH2 binding to p110α^15, 16, 20, 50, 51^. These interactions are dominated by electrostatic interactions where the predominantly negatively charged loop of p110α (residues 542–546) interacts with the positively charged nSH2 domain of p85α, positioning nSH2 in an inhibitory structure^15, 16, 18, 20, 51^. Our finding supports earlier studies indicating that mutations that increase the positive surface charge of p110α could lead to gain of function and suggesting that exon9 mutations in the helical domain of p110α protein may have a functional and possibly clinically relevant effect^16^. We note with interest that when stratifying by histology, the effect of exon9 AA^+^ mutations on survival appeared prevalent in patients of non-endometrioid histologic type, as survival was impaired significantly in this group (p = 0.019; Supplementary Fig. S1a, mutations observed in 8.8% of cases) but not within the endometrioid types (p = 0.426; Supplementary Fig. S1b, mutations observed in 4.4% of cases). Neither exon9 nor exon9 AA^+^ mutations were associated with non-endometrioid histologic type that as such could have confounded the survival analysis. Further studies, including more patients with charge-plus changing *PIK3CA* substitutions, in particular those with non-endometrioid tumors, and a better understanding of the molecular mechanism of p110α (and interaction partners) is required to confirm and extend our observations.

We did not find any correlation between the *PIK3CA* mutation status and clinicopathological variables in our data. This is in line with previous observations where no relation between *PIK3CA* mutations and clinical variables were found^24, 25, 35, 36, 45^ and *PIK3CA* mutations appeared randomly distributed within the recently suggested molecular subgroups of endometrial cancer^5^. In the literature, there is no reported association of *PIK3CA* mutations with age, histology, grade, FIGO stage, ER and PR status^24, 25, 36, 45^, although *PIK3CA* mutations have been associated with myometrial invasion depth^24^ and poor differentiation grade^25^. It is possible that analysis of the *PIK3CA* mutations in larger cohorts will reveal subgroups that might be useful in disease stratification and future treatment. Such subgroups could include critical regulators of the PI3K pathway, such *PIK3R1* and *PTEN*, again also found altered in endometrial cancer^5, 11^.

The finding of only one differentially expressed gene (RNF183) in our genome-wide array-based expression data, comparing tumors with and without *PIK3CA* mutations, is in line with the observed lack of associations with clinical and survival data we observe. Interestingly, the *RNF183* gene has previously been identified as a potential biomarker for detection of endometrial cancer in uterine aspirates^52^ and was also found to be an independent factor for the prediction of lymph node metastasis in this disease^53^.

Our sequencing strategy has some limitations. First, our overall level of detection is lesser than that what can be achieved by high throughput sequencing approaches, although comparable at hotspot sites (Supplementary Table S1). Second, as we choose to target exon9 and exon20 (encoding helical and kinase domains of p110α, including hotspot sites p.E542, p.E545, p.Q546 and p.H1047), the larger part of *PIK3CA* was excluded from the mutation screen. Previous data has indicated recurrent mutations within the amino-terminal of p110α located in exons 1–7 of *PIK3CA*, suggesting additional hotspot mutations such as the p.R88Q within exon 1^5, 17, 54^. Investigating N-terminal mutations (and other regions of p110α), and in particular their relation to clinical data, should be included in future studies; if not more high throughput approaches are selected that will cover this.

Metastases remains a key problem in oncology, as metastatic disease is responsible for up to 90% of cancer-related mortality, and yet one of the most poorly understood aspects of cancer^55^. *PIK3CA* mutations might predict response and resistance to targeted treatment with PI3K/AKT/mTOR pathway inhibitors as shown in clinical trials^56^. As a potential target, it is also relevant to explore the relationship of *PIK3CA* mutations between primary tissue and metastatic lesions, which is generally lesser explored due to lack of available tissue.

Our data show a concordance rate of 85.7% (6/7 cases) of specific mutations within our small sample set of matching primary tumor or metastases. The high consistency in our data seems to contradict the general heterogeneity issue revealed by high-throughput studies^41, 42^, but the homogeneity of *PIK3CA* mutations may particularly be attributed to two related observations. First, *PIK3CA* mutations do occur early in endometrial cancer progression, as shown earlier, and this may therefore contribute to the consistency of mutations we observe in our matched sample set^28, 43^. Second, *PIK3CA* mutations appear to be highly clonal in endometrial tumors as described in a recent high throughput sequencing study investigating spatial genetic relationships in the primary-metastasis setting^43^. A third explanation to the apparent “conserved state of *PIK3CA* mutations” is perhaps our focus on hotspot mutations in exon9 and −20, excluding mutation discoveries in other exons, and hence, possible masking the heterogeneity that might have been seen with other approaches. Bergström *et al*. ^57^ reported a concordance of 60% (6 of 10 cases) between paired samples of *PIK3CA* mutations in endometrial cancer. Focusing on their equivalent *PIK3CA* hotspot mutations (exons 9 and 20), they achieved a 57% concordance rate, which is somewhat lower compared to our study, although both studies display few paired-samples cases with *PIK3CA* mutations of either primary tumors, metastases or both. Thus, conclusions should be drawn with caution, and studies with larger set of paired primary tumors and metastases are warranted.

Our observation that *PIK3CA* mutations are present in the primary tumors and metastases with similar frequency, rules out the possibility that increased *PIK3CA* mutation rates in the metastases (rare mutations or a specific hotspot mutation) are “drivers” of the metastatic process itself. This emphasizes that increased knowledge about *PIK3CA* mutations, and their relation to other markers, is required to understand tumor initiation and progression, and for utilizing *PIK3CA* as a target in personalized therapeutic modalities

Investigation of the p-AKT/AKT-ratio in matched primary tumors and metastases showed no differences in expression levels. Thus, p-AKT levels may not be important in the metastatic progression, also underpinned by the association of increased tumor aggressiveness and decreased p-AKT/AKT-ratio (p = 0.003; Supplementary Fig. S3f), indicating that high p-AKT might be more important earlier during tumor development. On the other side, *PIK3CA* mutations seemed to activate AKT, as expression was higher among mutated primary and metastatic tumors (Supplementary Table S5 and Supplementary Fig. S3c), similar to observations made in colorectal cancer^58^. This suggests that *PIK3CA* mutations activate an AKT dependent pathway that, as suggested by Vasudevan *et al*.^59^, is most important in the formation of primary tumors.

*PIK3CA* mRNA expression increased from primary tumors to their corresponding metastasis (p = 0.031; Fig. 4a), also seen as a tendency of increased PI3K signature score (although only borderline significant p = 0.055; Fig. 4c). Previously, *PIK3CA* mRNA expression has been found to increase from normal control tissue to endometrial cancer tissues and from endometrioid to non-endometrioid histologic type^47^. Interestingly, in our study *PIK3CA* mutations seemed most likely to affect *PIK3CA* expression negatively (Fig. 4b and Supplementary Fig. S3a). We also found that high *PIK3CA* mRNA expression associated with poor prognosis and showed increased expression in metastases, suggesting independence of *PIK3CA* mutation status.

## Conclusion

In summary, this study suggests that exon9 mutations of *PIK3CA*, in particular those leading to charge-plus changing substitutions, could affect structural properties of the encoded protein p110α, and this may provide a functional link to the reduced survival observed in affected endometrial cancer patients. Moreover, *PIK3CA* mutations seem to be present in primary tumors and metastases at relatively high consistency, suggesting early occurrence and “genetic conservation” during progression towards metastases. Finally, our data supports several studies demonstrating that AKT-signaling may be both dependent and independent of *PIK3CA* mutations in cancer.

## Materials and Methods

### Patient series

Since May 2001, fresh frozen tumor samples from paired primary and metastatic lesions have been prospectively sampled together with clinical data and formalin-fixed paraffin-embedded (FFPE) tissues from endometrial carcinoma patients at the Department of Gynecology and Obstetrics, Haukeland University Hospital, Bergen, Norway. Fresh frozen primary tumors from 280 patients were subjected to molecular profiling. From this group of patients, 32 cases also had additional corresponding fresh frozen tissue available for metastatic lesions. In nine of these patients, several recurrences were available adding up to the total number of 45 metastatic lesions. In total, 532 patients were included in this study, with histologic types as described in Supplementary Table S6.

Primary treatment consisted of abdominal hysterectomy with bilateral salpingo-oophorectomy, unless surgery was deemed contraindicated. For the majority of the patients, the primary treatment also consisted of pelvic lymphadenectomy as a surgical staging procedure. Adjuvant therapy was recommended for patients with FIGO stages ≥ II and high-risk FIGO I patients, defined as non-endometrioid tumors or deeply infiltrating endometrioid grade 3 tumors, according to national guidelines and as previously reported^26^.

Comprehensive clinicopathologic data were available for all patients. Clinical data included patient age at diagnosis, International Federation of Gynecology and Obstetrics (FIGO) stage according to the 2009 criteria, histologic type and grade, lymph node status, myometrial infiltration, adjuvant/further treatment and follow-up informationMedian follow-up time was 59 months, last time point February 2017.

The study was approved by the Norwegian Data Inspectorate, Norwegian Social Sciences Data Services (15501), and the Western Regional Committee for Medical and Health Research Ethics (REK_2009/2315). All patients gave written informed consent. All experiments were performed in accordance with relevant guidelines and regulations.

### DNA sequencing

Mutations in *PIK3CA* exon9 and −20 from endometrial carcinomas were investigated by employing genomic DNA obtained from freshly frozen tissue samples. For metastatic samples, DNA was obtained by whole-genome amplified DNA (GenomePlex Complete WGA kit 2, Sigma-Aldrich, St. Louis, MO, USA) and used as template in PCR reactions to conserve the original material. Twenty-five ng genomic DNA was used as template in the PCR reactions together with 0.4 μM forward/reverse primers (Supplementary Table S7a,b) applying the Multiplex PCR kit or HotStarTaq *Plus* Master Mix kit (both Qiagen, Hilden, Germany). PCR products were run by agarose gel electrophoresis prior to treatment with ExoSAP-IT (USB, Cleveland, OH, USA) and direct Sanger sequencing (both directions) employing the BigDye Terminator Sequencing Kit version 3.1, and Applied Biosystems 3730XL Analyzer (Foster City, CA, USA). The sequencing chromatograms were visualized with eBioX version 1.5.1 (output examples of hotspot mutations shown in Supplementary Fig. S4). Previously published *KRAS* mutation data^27^ were compared with the new mutational data for 250 overlapping cases in the current study.

### Oligonucleotide DNA microarray and analysis

The RNA was extracted, obtained and hybridized as previously reported^28^. Briefly, RNA was hybridized to Agilent whole human genome microarray 44k (Cat.no G4112F, Santa Clara, CA, USA), scanned with Agilent Microarray Scanner Bundle. Median spot signals were employed as expression values, which were subsequently quantile normalized and log2-transformed. Expression data were available for 272 cases of endometrial cancer.

The PI3K pathway activation signature was based on the expression of 160 genes derived from *in vitro* cell line experiments^29^. When calculating the signature score for each sample, we subtracted the expression values of the down-regulated genes included in the signature, from the up-regulated genes. The expression values were mean normalized and scaled to the same standard deviation^30^.

Significance analysis of microarray (SAM) was performed to evaluate genes differentially expressed between various *PIK3CA* mutated and non-mutated groups by using J-Express^31^. We applied cutoff levels of False Discovery Rate (FDR) < 0.001, q-value < 0.001 and fold change ≥1.5 for calling significantly mutated genes.

For stratification of *PIK3CA* mRNA into “high/low expression”, the 5-year disease-free survival plots (Kaplan-Meier method) according to quartiles of *PIK3CA* mRNA expression were explored. Quartiles 1–3 clustered together with favorable prognosis and were defined as “low expression”. The 4^th^ quartile showed significant poorer survival compared to the rest, and was set to “high expression”.

### Fluorescent *in situ* hybridization (FISH)

FFPE tumor tissues from primary tumors (hysterectomy specimens) were mounted on tissue micro arrays (TMA) as previously described^27, 28^. The *PIK3CA* gene copy number was evaluated by FISH in TMAs using the Histology FISH Accessory Kit (Dako Agilent, Santa Clara, CA, USA) according to the manufacturer recommendations with minor modifications^28^. Slides were heated over night at 58 °C and deparaffinized for 3 × 10 min in xylene. De- and rehydrations were carried out in three steps with 100%, 85% and 70% ethanol before 15 min of pepsin digestion. The TMAs were incubated with *PIK3CA*-/CEP3 Dual Color probe (Abnova, Taipei City, Taiwan) at 75 °C for 5 min and 37 °C for approximately 72 hours. All spots from one case were screened (1–9 spots per patient) looking for copy number increases. Then, taking the whole area of each spot into account, the area with optimal signal quality and quantity to detect possible copy number increases was selected for assessment of gene and centromere signals in 20 non-overlapping nuclei. Status of *PIK3CA* gene copy levels were evaluated as “polysomy” (CN ≥ 2.3) or as “focal amplification” (*PIK3CA*/CEP3 probe ratio ≥1.15). Amplified cases were classified with polysomy only if not focally amplified.

### Immunohistochemical staining (IHC)

For detection of the p85α regulatory subunit of PI3K, TMAs were dewaxed in xylene, rehydrated in ethanol before microwave antigen retrieval at pH9 and incubation for 30 min with the mouse monoclonal antibody SC-1637 (Santa Cruz Biotechnology, Santa Cruz, CA), diluted 1:20. EnVision + System-HRP, anti-mouse was used as secondary antibody (Dako K4001). Evaluation of the staining index (index score 1–9), considering the product of staining intensity and area of maximal staining determined the level of staining^32^. Details, indexing and cut-off levels for IHC markers are summarized in Supplementary Table S8 ^60, 61^.

### Reverse Phase Protein Array (RPPA)

Protein levels of AKT (AKT1) and phosphorylated AKT (p-AKT_T308 and p-AKT_S473) were assessed by RPPA in 376 patients with endometrial cancer (p-AKT reported here as p-AKT_T308 only, as results were similar with p-AKT_S473^33^). The procedure was done as previously described^5, 34^. In brief, fresh frozen tumor samples were homogenized in lysis buffer followed by denaturation using SDS and subsequently serially diluted in lysis buffer. The lysates were printed on nitrocellulose coated slides before staining with RPPA-validated primary antibodies. The signal was captured by secondary antibodies, using a BioGenix autostainer and revealed by Dako Cytomation-catalyzed systems and DAB colorimetric reaction. Array-Pro analyzer (Media Cybernetics, Washington DC, USA) was used for quantifying spot signal intensities, before scanning of slides by CanoScan 9000F. Relative protein levels were determined by fitting each dilution curve with a logistic model (“Supercurve Fitting”; http://bioinformatics.mdanderson.org/OOMPA). Determination of cut-off in survival analysis for groups of high or low expression was performed as described for *PIK3CA* mRNA.

### Statistical analysis

Statistics was performed using Statistical Package of Social Sciences (SPSS), version 20.0 (IBM Inc., Chicago, IL). The *P-*values represent two-sided tests considered to be significant when less than 0.05. For evaluating associations between categorical variables, the Pearson’s *χ* ^2^-test was used. Mann-Whitney U-test was used for analysis of continuous variables between categories. One case with mutations in both exons9 and exon20 was excluded from the analysis correlating specific exon mutations to phenotype. When comparing exons, this double mutated case was categorized as being exon9 mutated due to presence of the p.Q546R hotspot mutation. Chi squared test was used to compare the mutation frequency between primary tumors without recurrence, primary tumors with recurrence and metastatic lesions, and also within the paired set of primary and corresponding metastatic lesions. Univariate survival analyses were performed using the Kaplan-Meier method, with date of primary surgery as entry date, and death specifically due to endometrial carcinoma (disease-specific survival) or no recurrence of endometrial cancer (recurrence-free survival) as end points. For estimation of differences in survival between groups the two-sided log-rank (Mantel-Cox) tests was used. The Cox proportional hazard regression model was employed to evaluate the prognostic impact of *PIK3CA* exon9 charge-plus changing substitutions (AA^+^) adjusted for other prognostic parameters including age, histologic type and grade, which are factors with prognostic impact in endometrial cancer.

## Electronic supplementary material

Supplementary_File
