# Supplementary material for: PIK3CA exon9 mutations associate with reduced survival, and are highly concordant between matching primary tumors and metastases in endometrial cancer
Source: Sci Rep. 2017 Aug 31;7:10240. doi: 10.1038/s41598-017-10717-z (PMC5578954; doi:10.1038/s41598-017-10717-z)
Supplement: Supplementary file 1 — Supplementary_File [file 41598_2017_10717_MOESM1_ESM.pdf]

Supplementary Information:

Mjos et.al.,

PIK3CA exon9 mutations associate with reduced survival, and are highly concordant between matching primary tumors and metastases in endometrial cancer.

Siv Mjos[1,2], Henrica M. J. Werner[1,2], Even Birkeland[3,4], Fredrik Holst[1,2], Anna Berg[1,2], Mari K. Halle[1,2], Ingvild L. Tangen[1,2], Kanthida Kusonmano[2,5,6], Karen K. Mauland[1,2], Anne M. Oyan[1,7], Karl-Henning Kalland[1,7], Aurélia E. Lewis[8], Gordon B. Mills[9], Camilla Krakstad[1,2], Jone Trovik[1,2], Helga B. Salvesen[1,2,†] & Erling A. Hoivik[1,2]*

*Corresponding author: Erling A. Hoivik, Centre for Cancer Biomarkers (CCBIO), Department of Clinical Science, Section for Gynecology and Obstetrics, University of Bergen, Jonas Lies Vei 72, 5020 Bergen, Norway. Phone: +47 5597 0723 Fax: +47 5597 4968. E-mail: erling.hoivik@uib.no (E.A.H.)

Supplementary Figure S1

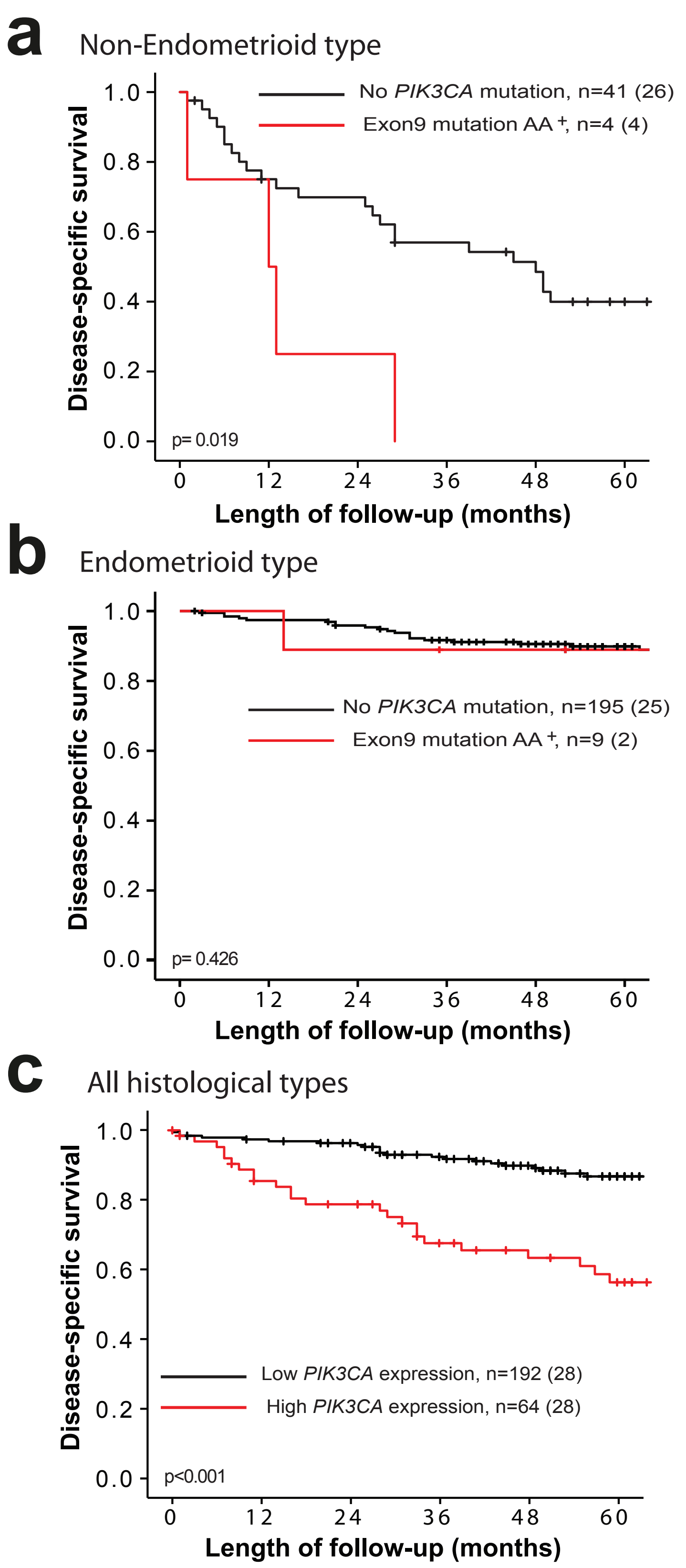

Supplementary Figure S1.

Disease-specific survival according to exon9 charge-plus mutations and *PIK3CA* mRNA levels in endometrial cancer.

(A) Within the non-endometrioid histologic type, exon9 charge-plus mutations of *PIK3CA* associate with poor survival, while they do not in the endometrioid type (B), as shown by Kaplan-Meier estimation plots. (C) High *PIK3CA* mRNA expression is associated with poor survival in endometrial cancer (all histologies) compared to the low expression group. n=number of cases (disease specific deaths). Categories are compared by log-rank (Mantel-Cox) test.

Supplementary Figure S2

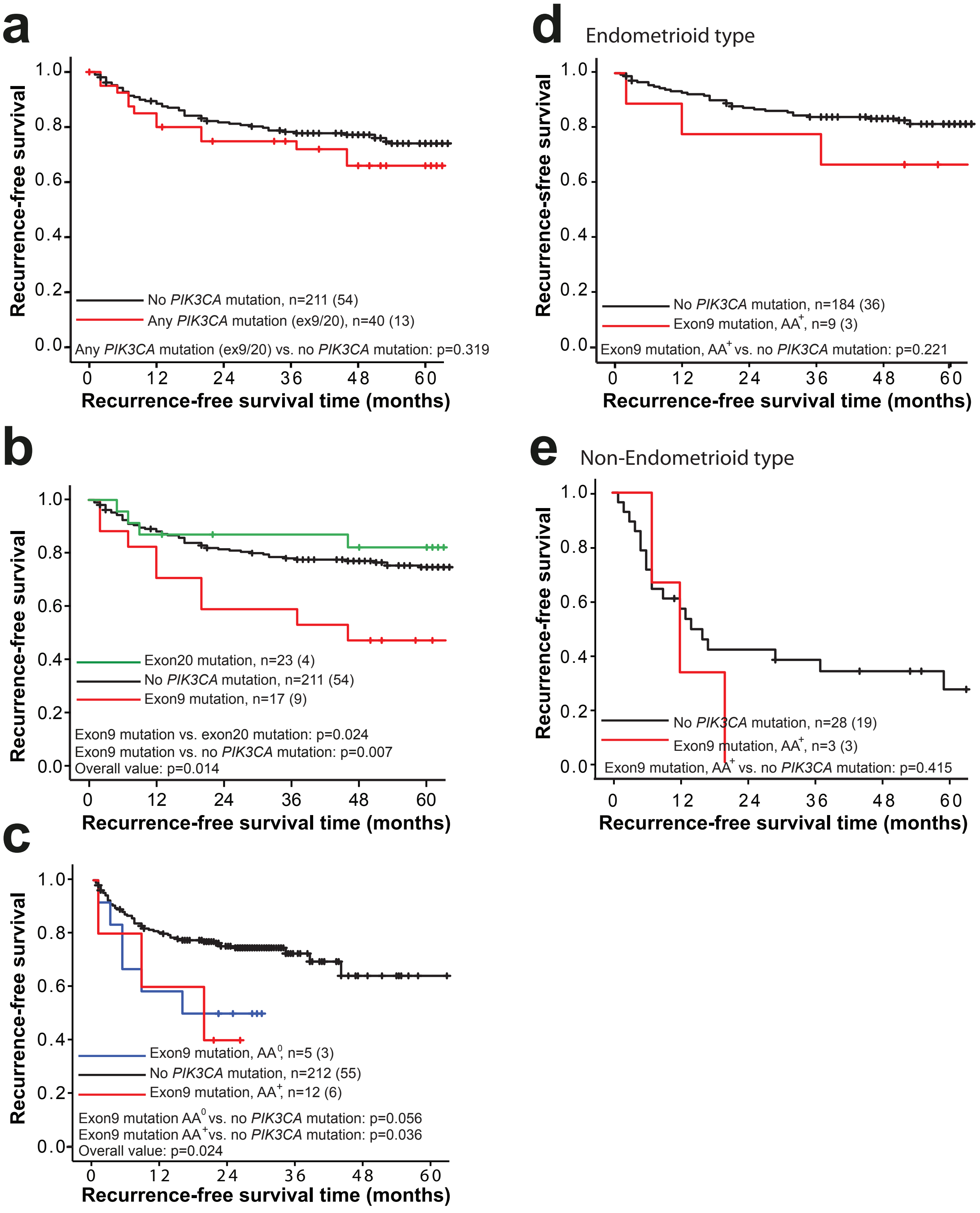

Supplementary Figure S2.

Recurrence-free survival according *PIK3CA* mutation staus in primary tumors of endometrial cancer.

Recurrence-free survival according to *PIK3CA* mutation status: (A) Any mutation; exon9 or exon20. (B) Mutations specifically in exon 9 or exon 20. (C) Mutations stratified as exon9 charge-plus or "neutral" substitution. (D-E) The effect on recurrence-free survival of exon9 charge-plus mutations within the histologic types endometrioid (D) and non-endometrioid (E). Curves are plotted by the Kaplan-Meier method; n = number of cases in each category followed by number of specific deaths to the among recurrence-free patients, with comparison of survival between categories using log-rank (Mantel-Cox) test.

Supplementary Figure S3

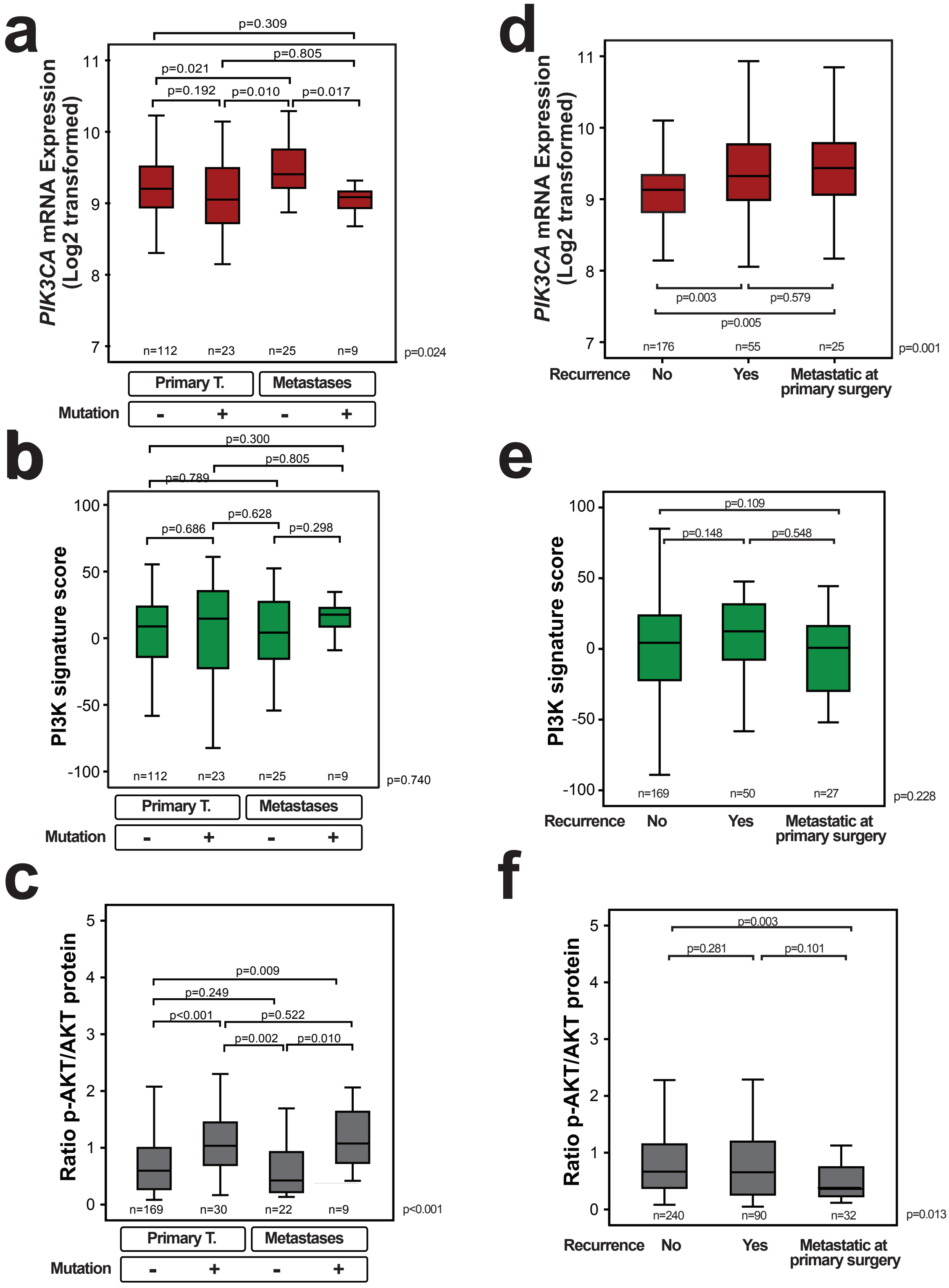

Supplementary Figure S3.

The effect of *PIK3CA* mutation in endometrial cancer in relation to disease progression measured by *PIK3CA* mRNA, p-AKT/AKT and PI3K activation signature score*.

PIK3CA mutation status compared to levels of *PIK3CA* mRNA expression, PI3K signature score, p-AKT/AKT-ratio of protein levels in primary tumors and metastases (A-C) or the status of systemic disease or recurrence (D-F). Statistical test were performed with Mann-Whitney U test for pairwise comparison, and Kruskal-Wallis test when more than two groups were compared. *The PI3K mRNA signature score defined by Gustafson *et al.* [29].

Supplementary Figure S4

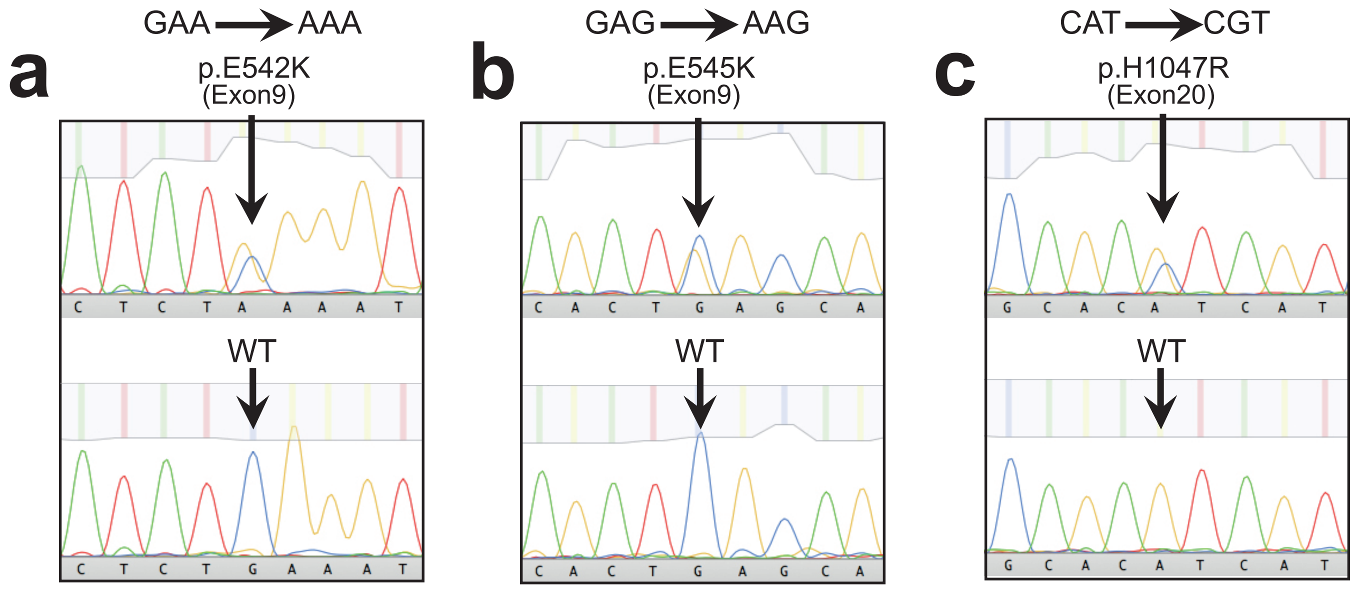

Supplementary Figure S4.
Examples of *PIK3CA* hotspot mutations in endometrial carcinomas detected by Sanger sequencing.
(A-C) Hotspot mutations of p.E542K, p.E545K and p.H1047R shown as sequencing chromatograms, with mutated nucleotide indicated.

Supplementary Table S1.

Comparing reported frequencies of *PIK3CA* mutations in endometrial cancers studies.

PIK3CA mutations	**Percent of mutated hotspot positions within the exons**			**Mutation frequency exon9 and -20**	**Overall mutation frequency of *PIK3CA***	**Number of cases**	**Sequencing Method**	**References**
	**Exon9**	**Exon20**	**Both exons**					
**This study**	94.7%	60.0%	75.0%	15.7%	Not assessed	281	Sanger	This study
**TCGA Uterine Corpus Endometroid Carcinoma**	97.4%	51.3%	74.4%	31.5%	53.2%	248	WES	Refs. [5,13,14]
**COSMIC Endometrium Carcinomas**	96.3%	61.8%	75.0%	14.0%	22%	2989	WES	Ref. [39]

Abbreviations: Sanger; Sanger sequencing, WES; Whole exome sequencing. TCGA; The Cancer Genome Atlas (accessed through cBioPortal http://www.cbioportal.org). COSMIC; Catalogue of Somatic Mutations in Cancer, v79 (http://cancer.sanger.ac.uk/cosmic).

Supplementary Table S2.

***PIK3CA* mutations detected in exon9 (helical domain) and exon20 (kinase domain) in endometrial carcinomas.**

PIK3CA mutations						
Alteration type					n observed	
Exon	cDNA[a]	Amino Acid alteration	Hot-spot site	Exon9 charge[b]	PT	M
9	**c.1618C>G**	**p.L540V**	no	0	1	
9	c.1624G>A	p.E542K	yes	+	4	1
9	**c.1624G>T**	**p.E542*(STOP)**	yes	0	1	
9	c.1625A>T	p.E542V	yes	0	1	
9	c.1633G>A	p.E545K	yes	+	5	5
9	c.1634A>C	p.E545A	yes	0	1	
9	c.1634A>G	p.E545G	yes	0	2	
9	c.1635G>T	p.E545D	yes	0	1	
9	c.1636C>A	p.Q546K	yes	+	2	1
9	c.1636C>G	p.Q546E	yes	0	1	
9	c.1637A>G	p.Q546R	yes	+	1	1
9	c.1638G>C	p.Q546H	yes	+	1	
20	c.3012G>T	p.M1004I	no		1	
20	c.3073A>G	p.T1025A	no		2	
20	c.3103G>A	p.A1035T	no		1	
20	c.3104C>T	p.A1035V	no		1	
20	c.3129G>A	p.M1043I	no		1	
20	c.3129G>T	p.M1043I	no		1	
20	c.3131A>G	p.N1044S	no		1	
20	c.3132T>G	p.N1044K	no		1	
20	c.3140A>G	p.H1047R	yes		13	1
20	c.3140A>T	p.H1047L	yes		1	
20	c.3141T>G	p.H1047Q	yes		1	1
20	c.3145G>A	p.G1049S	no		1	1
20	**c.3148G>A**	**p.G1050S**	no		1	
Total number of analyzed tumor samples					281	53

Alterations in bold indicates *PIK3CA* mutations not previously reported in endometrial cancer according to cBioPortal (http://www.cbioportal.org/; The Cancer Genome Atlas database) or the COSMIC (http://cancer.sanger.ac.uk/cosmic; Catalogue of Somatic Mutations in Cancer, v79) both accessed Feb, 2017). Metastatic lesions; M and primary tumors; PT. 23 PTs had one corresponding metastatic lesion, five patients had two metastatic lesions and four patients had three metastatic lesions. For seven patients, only the metastatic lesion was available and of these patients, one had two metastatic lesions accessible. [a]Nomenclature according to www.hgvs.org/mutnomen/. [b]Resulting charge of amino acid substitution; + symbolize mutations predicted to cause an electrostatic charge reversal of the amino acid from negative to positive, 0 symbolize mutations not predicted to cause increased positive charge of the amino acid.

Supplementary Table S3. Comparison of clinico-pathologic variables and *PIK3CA* mutation type in primary endometrial carcinomas assessed by Sanger sequencing.

PIK3CA mutation status		No mutation	*PIK3CA* mutation type			
			exon9 mutation		exon9_charge-plus mutation	
**Variable**	***Total cases, n***	**n (%) ex9/charge**	**n (%)**	**p-value[a]**	**n (%)**	**p-value[a]**
Age	255/237			0.542		0.891
< 66		123 (93.9/94.6)	8 (6.1)		7 (5.4)	
≥ 66		114 (91.9/95.0)	10 (8.1)		6 (5.0)	
BMI	247/229			0.638		0.233
< 30		153 (92.2/93.3)	13 (7.8)		11 (6.7)	
≥ 30		76 (93.8/97.4)	5 (6.2)		2 (2.6)	
FIGO stage	255/237			0.370		0.300
I-II		191 (93.6/95.5)	13 (6.4)		9 (4.5)	
III-IV		46 (90.2 /92.0)	5 (9.8)		4 (8.0)	
Myometrial infiltration	253/236			0.606		1.000
<50% infiltration		140 (94.0 /95.2)	9 (6.0)		7 (4.8)	
≥50% infiltration		96 (92.3/95.0)	8 (7.7)		5 (5.0)	
Lymph node metastasis	212/198			1.000		0.661
No		170 (92.3/95.0)	12 (6.6)		9 (5.0)	
Yes		28 (93.3/93.3)	2 (6.7)		5 (6.7)	
Histologic type	255/237			0.749		0.267
Endometrioid		195 (93.3/95.6)	14 (6.7)		9 (4.4)	
Non-Endometrioid		42 (91.3)	4 (8.7)		4 (8.7)	
Grade	253/235			0.314		1.000
High-medium		149 (94.3/94.9)	9 (5.7)		8 (5.1)	
Low		86 (90.5/94.5)	9 (9.5)		5 (5.5)	
DNA ploidy	210/205			0.204		0.695
Diploid		147 (94.8/95.6)	8 (5.2)		6 (3.9)	
Aneuploid		49 (89.1/94.2)	6 (10.9)		3 (5.8)	
ERα	248/231			0.574		0.497
Positive		175 (93.6/95.6)	12 (6.4)		8 (4.4)	
Negative		56 (91.8/93.3)	5 (8.2)		4 (6.7)	
PR	251/234			0.573		1.000
Positive		174 (92.6/95.1)	14 (7.4)		9 (4.9)	
Negative		60 (95.2/95.1)	3 (4.8)		3 (4.8)	

[a]The p-value was estimated using the Chi-square tests (Pearson or Fisher`s exact test). n= number of cases analyzed. Abbreviations: BMI; body mass index, FIGO; International Federation of Gynecology and Obstetrics.

Supplementary Table S4
Multivariable survival analysis of 246 endometrial cancer patients according to Cox` proportional hazards regression model.

Variable	n	Unadj.HR	95% CI	p-value	Adj.HR	95% CI	p-value
**Age (mean=65)**	246		1.02-1.07	**<0.001**	1.03	1.32-4.78	**0.026**
**Histologic grade**				**<0.001**			**<0.001**
grade 1-2	157	1			1		
grade 3	89	7.72	4.22-14.13		4.41	2.09-9.29	
**Histologic type**				**<0.001**			**0.005**
EEC	201	1			1		
NEEC	45	7.66	4.56-12.87		2.51	1.32-4.78	
***PIK3CA* MUT status**				**0.023**			**0.012**
noMUT	233	1			1		
ex9charge-plus MUT (AA$^+$)	13	2.68	1.15-6.26		3.00	1.27-7.07	

Supplementary Table S5.
Correlation between mutations, amplifications and mRNA expression of *PIK3CA*, and *KRAS* mutations, and p85, PTEN, Stathmin and p-AKT(T308) expression in endometrial carcinomas.

Variable	Method (total n)	No *PIK3CA* mutation n (%)	Any *PIK3CA* [a] mutation n (%)	p-value[b]
***KRAS* gene**	Sanger			0.618
Non-mutated	sequencing	180 (84.1)	34 (15.9)	
Mutated	(n=250)	32 (88.9)	4 (11.1)	
***PIK3CA* gene amplification**[c]	FISH (n=234)			0.642
Polysomy		16 (76.2)	5 (23.8)	
Focal		24 (82.8)	5 (17.2)	
Not amplified		155 (84.2)	29 (15.8)	
**p85α expression**	IHC			0.559
Low	(n=236)	106 (86.9)	16 (13.1)	
High		96 (84.2)	18 (15.8)	
**PTEN expression**	IHC			0.884
Low	(n=256)	51 (85.0)	9 (15.0)	
Middle		102 (85.7)	17 (14.3)	
High		64 (83.1)	13 (16.9)	
**Stathmin expression**	IHC (n=258)			0.562
Low		182 (86.3)	29 (13.7)	
High		39 (83.0)	8 (17.0)	
***PIK3CA* mRNA**	Microarray			0.075
Low	(n=169)	66 (75.9)	21 (24.1)	
High		71 (86.6)	11 (13.4)	
**p110α**	RPPA	87 (87.9)	12 (12.1)	0.242
Low	(n=200)	82 (81.2)	19 (18.8)	
High				
**p-AKT/AKT**	RPPA			0.007
Low	(n=199)	50 (96.2)	2 (3.8)	
High		119 (81.0)	28 (19.0)	

[a]PIK3CA mutation status by exon9 and -20. [b]Chi-square test or Fisher's Exact Test when less than five patients in a group. [C]Copy number increase (CN) set to; focal amplification (ratio≥1.15) or polysomy (CN≥2.3, or increase not focal). Abbreviations: FISH; fluorescence in situ hybridization, IHC; Immunohistochemistry, RPPA; reverse phase protein array, p-AKT; phosphorylated AKT at amino acid T308.

Supplementary Table S6. Overview of the histologic types of primary endometrial cancer samples included in this study.

Histologic type, by two groups	Specific histology type	All cases in cohort, any assay (%)	Cases with sequencing data (%)	Cases with exon9 charge-plus mutations (%)
**Endometrioid**	Endometrioid	439 (82.5)	224 (80.0)	9 (69.2)
	Adenosquamous	3 (0.6)	2 (0.7)	-
**Non-Endometrioid**	Clear cell	14 (2.6)	8 (2.9)	-
	Serous	44 (8.3)	25 (8.9)	3 (23.1)
	Carcinosarcoma	23 (4.3)	14 (5.0)	1 (7.7)
	Undifferentiated	9 (1.7)	7 (2.5)	-
**Total numbers**		n=532 (100.0)	n=280 (100.0)	n=13 (100.0)

Supplementary Table S7a.
Oligonucleotides used as primers for PCR amplification and direct sequencing of *PIK3CA* exon 9 and exon 20 targets.

Exon	Primer (5'-3')	Reverse primer (5'-3')
Primers for PCR amplification		
9	GGGAAAAATATGACAAAGAAAGC	CTGAGATCAGCCAAATTCAGTT
20	GCTCCAAACTGACCA	TGGAATCCAGAGTGAGCTTTC
Primers for Sanger sequencing		
9	GGAAAAATATGACAAAGAAAGCTATATAAG	ACAGAGAATCTCCATTTTAGCAC
20	CTCAATGATGCTTGGCTCTG	TGGAATCCAGAGTGAGCTTTC

Supplementary Table S7b.
PCR cycling conditions:

Step	Duration time	Temperature
Initial activation /denaturation	5 min/ 15 min[a]	95°C
1. Denaturation	45 s	94°C
2. Annealing	90 s	60°C
3. Elongation	90 s	72°C
Repeat step 1-3 for additional 38 cycles		
Final elongation	90 s	72°C

[a] Time adjusted for Multiplex PCR or HotStarTaq *Plus* Master Mix PCR as described in Qiagen protocols.

Supplementary Table S8.
Protein expression assessed by IHC: antibodies, staining and evaluation of proteins analyzed in this study.

Target antigen	Antibody Name	Vendor (cat.num.)	Antibody ID[a]	Clonality (species)	Clone ID	Staining index[b] groups and evaluation	References
**p85α**	PI3-kinase p85α (B-9) antibody	Santa Cruz Bio-technology (sc-1637)	RRID:AB_628126	monoclonal (mouse)	B9	Groups by SI; low (1-2), high (3-4).	This study
**Estrogen Receptor α**	Estrogen Receptor α antibody	Dako (M7047)	RRID:AB_2101946	monoclonal (mouse)	1D5	Groups by SI; low (0-3), high (4-9).	Ref.[32]
**PTEN**	PTEN (D4.3) XP Rabbit mAb antibody	Cell Signaling Technology (9188)	RRID:AB_2253290	monoclonal (rabbit)	D4.3	Groups by SI; low (0), intermediate (1-4), high (6-9).	Ref.[60]
**Progesterone Receptor**	Progesterone Receptor antibody	Dako (M3569)	RRID:AB_2532076	monoclonal (mouse)	PgR 636	Groups by SI; negative (0), positive (1-9).	Ref.[61]
**Stathmin**	Stathmin antibody	Cell Signaling Technology (3352)	RRID:AB_330234	Polyclonal (rabbit)	n.a	Groups by SI; low (0-6), high (9).	Ref.[26]

[a]RRID; unique antibody identifier http://antibodyregistry.org/. [b]The staining index (SI); the product of the staining intensity (0-3) and area of positive tumor cells (1≤10%, 2=10–50% and 3≥50%), was used for semi-quantitative evaluation.
